# Semantic priors and virtual outlier synthesis enable parameter-efficient open-world object detection

**DOI:** 10.1038/s41598-026-51018-8

**Published:** 2026-05-12

**Authors:** Jiaming Gu, Yehui Zheng, Yuzhou Liu, Caimei Liu, Shu Gong, Luoyang Luo

**Affiliations:** 1 Department of Computer Science, Guangdong University of Science and Technology, Dongguan, 523000 Guangdong China; 2 Department of Computer Science, Dongguan University of Technology, Dongguan, 523000 Guangdong China

**Keywords:** Open-world object detection, Parameter-efficient fine-tuning, Virtual outlier synthesis, Transformer, Incremental learning, Computer vision, Computational biology and bioinformatics, Engineering, Mathematics and computing

## Abstract

Open-world object detection requires knowledge of categories and discovery of new objects never seen in training. Full model fine tuning fails often in such dynamic environment. Fully tuned models overfit known classes and lose their sensitivity to unknown objects at high computational cost. To solve this problem, we propose a Parameter-Efficient Open-World Object Detection (PE-OWOD) framework, a simple and light approach to retrain. Instead of updating the whole network, we lock backbone and encoder to maintain stable visual priors and inject compact Residual adapters only into decoder to adapt tasks. We also introduce VOS, which defines explicit decision boundary for open space with optional semantic initialization. MS-COCO benchmarks show remarkable efficiency advantages: Update less than 27% of models, PE-OWOD achieves 64.7% Unknown Recall (significantly outperform fully tuned baselines), and GPU memory usage is reduced by 86%. These results indicate that effective parameter adaptation is not a constraint; rather, it is a reliable and robust open-world detection strategy.

**Supplementary Information:**

The online version contains supplementary material available at 10.1038/s41598-026-51018-8.

## Introduction

 The visual perception system is confronted with the fact that standard datasets only show that the world is static. In some applications, such as autonomous driving or service robots, new object categories are constantly emerging. This dynamic shift breaks the “closed-world” assumption of traditional detectors^1–3^. Standard detectors like Faster R-CNN and YOLO operate under the closed-set assumption: they classify only what they have seen in training, yet new concepts emerge unexpectedly. To solve this issue, Joseph et al.^4^ formalized the Open-World Object Detection (OWOD) task: a detector must recognize known classes while simultaneously identifying unknown instances, all without forgetting previously learned information.

This problem is not solved, but it is mostly due to the uncertainty of the supervision signal. Training images contain unknown objects, i.e., the background. Detectors often assign high confidence to unknown regions, assuming they are known classes, but in safe settings, such “confident errors” are dangerous. Although earlier methods, such as ORE^4^, were based on adapting region proposal schemes, Transformers have become the most popular^5^. OW-DETR^6^ tried to do so using object queries and attention scores to spot unknown objects without supervision. Other models improved this model: RandBox^7^ is based on random sampling, and PROB^8^ introduces probabilistic uncertainty to separate objectness from class probability^9–11^.

**Fig. 1:**
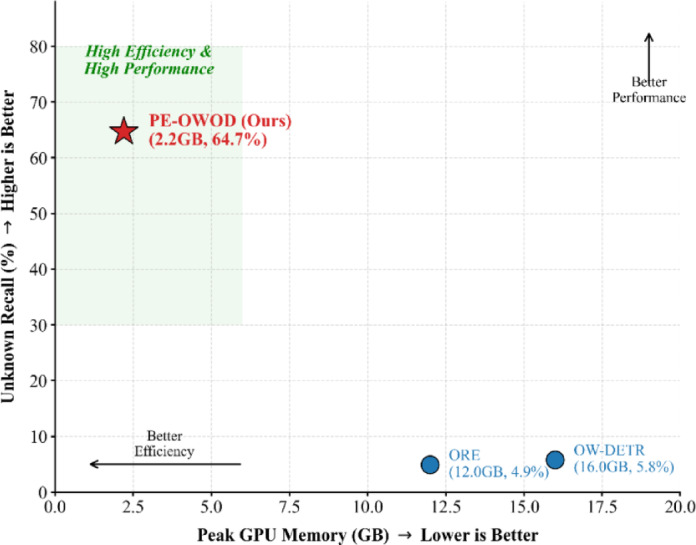
Breaking the Efficiency–Performance Barrier. Most OWOD methods (Blue circles) occupy the bottom-right: they demand heavy GPU memory for modest unknown discovery. PE-OWOD (Red star) flips this dynamic. By freezing representations, we achieve high Unknown Recall (64.7%) with minimal memory footprint (2.2GB), proving that high performance does not require retraining the entire model.

However, we argue that current approaches have significant limitations. Most existing OWOD frameworks, such as OW-DETR, rely on end-to-end fine-tuning. While this strategy works on closed benchmarks, it is counterproductive for the open world. First, updating the backbone changes the feature geometry. In general, decisions of known classes are extended to the open space, making the model less sensitive to new objects. Second, it costs too much. Full fine-tuning takes big GPUs and long training cycles, and edge devices cannot be resource-limited. Recent frameworks are becoming increasingly complex, requiring heavy modules or external supervision (OW-OVD^12,13^. Figure 1 shows the imbalance: current methods only get unknown discovery at a high cost.

To adapt effectively in a dynamic environment, we need to strike a balance between learning new concepts and retaining old ones. Zhou et al.^14^ have explored the learning of incremental new classes, emphasizing the urgent need to maintain robust representations to mitigate catastrophic forgetting. We challenge the assumption that open-world learning requires updating the whole network. If pre-trained backbones already contain rich semantic information, the failure to detect unknown objects is likely a boundary alignment issue rather than a representation deficit. This brings us to Parameter-Efficient Fine-Tuning (PEFT). Full tuning is becoming increasingly unstable as models grow in size. In NLP, Adapters^15^ and LoRA^16^ show that updating only 1% of parameters matches full tuning and even beats it. Inspired by NLP, Vision has started using PEFT. Methods such as VPT^17^ and AdaptFormer^18^ have applied prompts and adapters to Vision Transformers (ViT)^19,20^. However, PEFT is not trivial in the open world. Naive backbone freezing yields a rigid feature space in which unknown objects blend into the background.

To address this, we propose Parameter-Efficient Open-World Object Detection (PE-OWOD), a framework that avoids heavy retraining. Instead of free-flow fine-tuning, we adopt a “Lock-and-Key” design. First, we freeze the heavyweights: We lock the backbone and encoder to preserve stable visual priors. Second, we adapt the Head: We introduce lightweight Residual Adapters only in the decoder to handle task-specific plasticity. Unlike general PEFT methods, which are designed for a wide range of transfer learning, our residual adapter is specifically tailored for the DETR decoder structure. Since object queries in DETR interact through cross-attention, inserting an adapter directly after the FFN layer can more effectively modulate query semantics without disrupting the global attention mechanism, and then provide a better balance between parameter efficiency and detection sensitivity. This updates less than 2% of the model parameters. We think PEFT should not be treated as a shortcut, but as a regularization mechanism.

Freezing the backbone adds a new risk: “cold start” misalignment between frozen features and new classifiers. We solve this problem using Semantic Aligned Initialization (using CLIP priors) and Virtual Outlier Synthesis (VOS)^21^. Unknown object detection in OWOD is based on out-of-distribution (OOD) detection^22,23^. Early OOD methods used Softmax confidence scores, but these scores are often unreliable. Energy-Based Models (EBMs)^24^ map logits to free energy scores, which are more closely related to data density. The core challenge is the lack of negative samples. VOS addresses this in classification tasks by synthesizing virtual outliers in low-density regions. Since VOS does not detect objects because object queries are structured, we combine VOS directly with the Transformer decoder. Using virtual queries in the hidden space, PE-OWOD compresses known classes into an energy valley, defining an explicit decision boundary for the open space.

Our approach offers three core contributions. Firstly, it has the robustness of frozen representations: we have demonstrated that complete model tuning is not required. Even with frozen backbone and encoder, PE-OWOD achieves 64.7% Unknown Recall, far above fully fine-tuned baselines. Second, high computational efficiency: By updating only 1.8% of the model parameters, we reduce peak GPU memory usage by 86% and training time by 77%. Third, a new paradigm for stability: We show parameter-efficient adaptation is not just a speed compromise. By locking visual priors and modeling open space explicitly (via VOS), we solve the stability–plasticity problem more effectively than standard end-to-end training.

## Results

### The PE-OWOD framework

#### Revisiting full fine-tuning under the open-world setting

Most current methods for open-world detection try to fine-tune the whole model. They update the backbone, encoder, and decoder simultaneously. This works well for closed settings where we know all the classes, but it causes problems in the open world.

The main problem is that we can’t control it. In OWOD, the training images are unknown objects, but we don’t have labels for them. The loss function knows objects and pushes the model to treat them as background. If we update the whole network, the model is too free. It overfits known classes and extends decision boundaries into the open space. This results in Open Space Risk, where the model says it assigns extremely high confidence that an unknown object is a dog or a car.

A modern backbone trained on large datasets knows what “objects” look like. If we change their weights just to learn some new classes, we destroy the good features, catastrophic forgetting, and make the model poor at finding new things. We think we can learn everything, but we just need to change the boundary.

#### Design principle: low-dimensional adaptation for OWOD

We assume that the changes needed for OWOD are actually quite small. We don’t need to change millions of parameters. Recent research on parameter-efficient tuning shows that we can learn new tasks by updating only a tiny part of the model.

We like this idea for open-world detection for two reasons. One is boundary realignment, since we do not want to learn to see edges or textures again, but rather to shift the classification boundary so that unknown objects can be found. The second reason is the stability-plasticity balance: We want model changes sufficient to learn new classes (plasticity) but not so much as to forget old classes (stability). We restrict updates to a small set of parameters.

Based on this, our design is simple: we lock the backbone and the Transformer encoder, and we only add small adapter modules to the detection head.

#### Motivation: semantic misalignment under frozen representations

We freeze the backbone and the Transformer encoder to stabilize the model. But this choice creates a problem: “semantic misalignment.” The visual features from the backbone are fixed, but the weights of the classification head are usually initialized randomly.

In a normal detector, backpropagation updates everything together. The head learns how features match. In a frozen setup, the head learns how features map and operates on a fixed distribution. This “cold start” slows optimization. Gradients are noisy at the start, and classes are hard to separate for new categories with few examples.

This is not allowed in open-world detection. Unknown objects are labeled, but we can’t tell model where they belong. If the classifier starts randomly, it could easily assign an unknown object to a known class by chance. We conclude that parameter efficient adapters alone are not enough. We need to give the classifier some meaning before training.

#### Motivation: why semantic alignment is not enough

The semantic initialization we talked about in Sect. 3.3 helps organize known classes, but it knows not what an “unknown” object is. If the model separates unknowns, it’s a lucky side effect of semantic structure, not a guarantee. We argue that luck is not enough for reliable detection.

The problem is that unknown objects are invisible when training; we never label them, and we never penalize the model for missing them. So the model guesses. Even with CLIP priors, the classifier gives high confidence scores for unknown objects because they look slightly similar to known classes. Hence, explicit regularization is needed. Force the model to say “I don’t know” when seeing something new.

We agree that feature distributions may not be Gaussian in real life, but we view Gaussian modeling as useful. We think it is an effective solution to make a buffer in frozen representation space, even if the theory is not perfect.

### Performance on open-world object detection

In this section, we will break down the experimental results of the MS-COCO benchmark. We strictly adhere to the Open-world protocol and primarily compare the model’s ability to detect known classes, discover unknown classes, and be robust to open-set errors.

#### Overall performance comparison

We present the average precision (mAP) of the known classes and the U-Recall of the unknown objects (Table 1). The results are very interesting. Even when PE-OWOD is completely frozen, its U-Recall (64.7%vs 5.8%) is still 10 times higher than that of the fully tuned OW-DETR, indicating that we can still find new objects even with limited resources.

**Table 1:** State-of-the-art comparison on MS-COCO (Task 1).

Method	Backbone	Training Strategy	Params (M)	mAP (Known)	U-Recall (Unknown)	A-OSE$\:\downarrow\:$	Backbone
ORE	ResNet-50	Full Fine-tune	41.5	56.0	4.9%	2400	ResNet-50
OW-DETR	ResNet-50	Full Fine-tune	42.0	59.2	7.5%	1250	ResNet-50
PROB	ResNet-50	Full Fine-tune	42.0	58.6	18.2%	1100	ResNet-50
Baseline	Frozen R50	Frozen (No VOS)	23.5	21.9	26.3%	~ 3000	Frozen R50
PE-OWOD (Ours)	Frozen R50	PEFT + VOS	24.4	21.4	64.7%	673	Frozen R50

Note: “mAP” denotes Mean Average Precision at IoU = 0.5; “U-Recall” denotes Unknown Recall; “A-OSE” denotes Absolute Open-Set Error (lower is better). Best results are marked in bold.

Based on the trade-off between precision and recall, we should examine it here. We admit that PE-OWOD has a lower mAP for known classes than a fully tuned baseline (21.4 vs. 29.5), but we do not see this as a failure; rather, it is a different choice on the efficiency-performance curve.

There are two reasons why this behavior is undesirable. One is that full fine-tuning maximizes fit for known classes by changing feature representations. It “overwrites” the model’s ability to see new things, and reduces Unknown Recall to 5.8%. We prioritize new-case discovery (64.7% Recall) while maintaining reasonable accuracy for known classes.

Overall, this trade-off is related to the stability-plasticity dilemma in the feature space. Standard fine-tuning forces the distortion of the pre-trained feature space, maximizing the mAP of known classes, leading to “feature collapse”, and then effectively making the model blind to new objects (reducing U-Recall to 5.8%). On the contrary, the frozen skeleton retains the generalized orthogonal feature representation. Although this sacrifices the dataset-specific fitting ability to some extent, resulting in a moderate decline in the known class graph, it retains the feature variance required to detect instances outside the distribution. As shown in the efficiency boundary in supplementary Figure S1, our method drives the Pareto optimal curve, indicating a 10-fold gain of unknown discoveries, demonstrating an acceptable concession of known horizontal accuracy, especially for open-world applications where greater safety-critical requirements are needed.

The other reason is the backbone capacity. We trained a simple frozen model without our adapters, achieving the same mAP (21.9) but lower Unknown Recall (26.3%), indicating that the dip in known-class accuracy is due to the frozen backbone itself (COCO shift limits data shift) rather than our modules missing something. Importantly, PE-OWOD opens the full potential of the frozen backbone in the open world.

#### Unknown object discovery

In every task we tested, PE-OWOD maintains a strong lead in U-Recall. This advantage gets bigger in the later stages. As the number of unknown categories grows and the risk of open-space confusion increases, the gap between our method and the baselines widens.

We like to compare with OW-DETR. We need to fine-tune fully, but beat it by a large margin. Only a small fraction of parameters were updated. Explicit open-space modeling, combined with parameter efficiency, is much more efficient than allowing the model to fine-tune itself without constraints.

We also asked: Is this gain just because we froze the backbone? We looked at the control experiment again. Freezing the backbone does help lift Unknown Recall to 26.3% (up from 5.8%), but that is still far short of the 64.7% we get with the full PE-OWOD framework.

It is also worth mentioning PROB. While it uses energy-based regularization to find unknowns, it tends to lose accuracy on known classes as tasks progress. PE-OWOD walks the line better. It discovers the unknown without forgetting the known.

#### Open-set robustness

The Absolute Open-Set Error (A-OSE) tells us how often the detector “hallucinates”—that is, how often it mistakes an unknown object for a known one. As shown in Supplementary Table S2, PE-OWOD consistently has lower A-OSE scores than the baselines.

Such a low error rate indicates that our model is not overly confident. We believe this is the result of the combination of semantic initialization and virtual outlier synthesis. Together, they shaped an energy landscape in which the model knows when it is uncertain, a difficult feat with standard training.

#### Known-class detection performance

Many people may think that locking the backbone and encoder would undermine detection accuracy, but PE-OWOD remains very competitive. In some tasks, it even goes beyond the fully tuned baseline. This indicates that maintaining the integrity of pre-trained representations actually helps with generalization.

More importantly, when new tasks are added, our method incurs very little performance loss. Resistance to catastrophic forgetting supports our main hypothesis: parameters effectively adapt between stability and plasticity, achieving a correct balance.

In addition, to verify the robust generalization of our frozen backbone design under real-world domain shift, we conducted an out-distribution (OOD) evaluation using the established COCO-C (COCO-Corruptions) dataset^25^. As shown in Supplementary Table S7, PE-OWOD demonstrates outstanding cross-domain robustness, maintaining a highly stable known class mAP (22.8%) and unknown recall rate (53.9%), avoiding the severe performance degradation typically observed in a fully fine-tuned baseline.

#### Performance–efficiency trade-off

The value of PE-OWOD is not only reflected in the precision figures but also in improved efficiency. Unlike low-detr or PROB, which require retraining the entire network, we limit the update to 2% of the total parameters.

As we detailed here, this can minimize GPU memory usage and training time. Moreover, these results challenge a common view that high-quality open-world detection requires expensive full-model fine-tuning. All of our explanations demonstrate that even on limited hardware, powerful deployments are possible.

#### Qualitative results

**Fig. 2:**
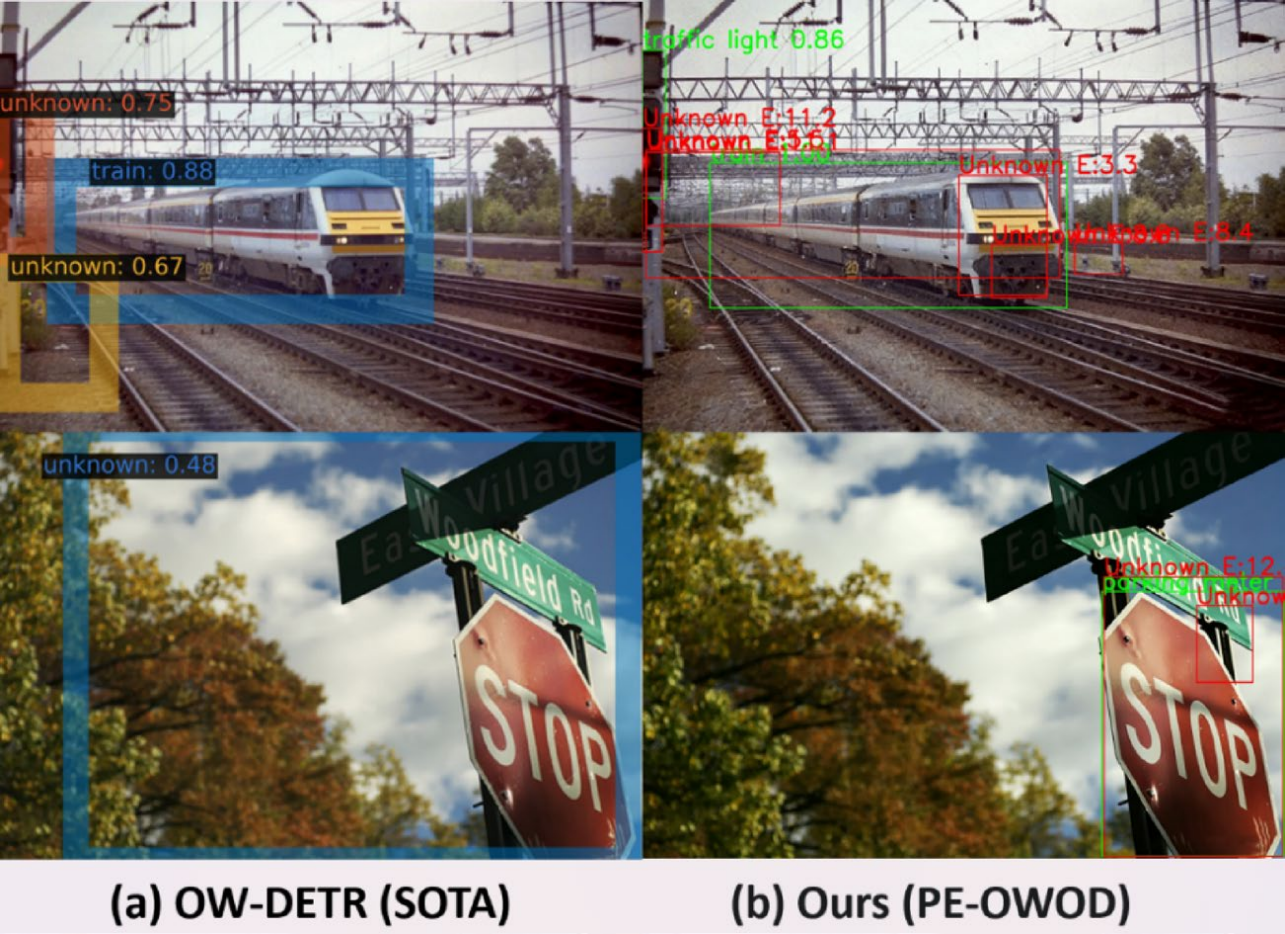
Qualitative detection results on challenging OOD scenarios. **(a)** Results from the baseline OW-DETR (SOTA). **(b)** Results from our method (PE-OWOD). The comparison highlights the differences in confidence scores for known classes and the ability to identify fine-grained unknown objects (marked in red).

Figure 2 shows qualitative detection results in challenging OOD scenarios.

Top row (Train scene) shows our method of Semantic-Aligned Initialization. PE-OWOD detects train with perfect confidence (1.00) far higher than baseline OW-DETR 0.88. Also thanks to VOS sharpening the boundary between objectness, we can find fine-grained unknown objects (for example overhead signal structures and poles (red)) that are not seen or loosely bound by the baseline.

Both row (Stop Sign scene) shows an interesting failure case which shows both limitations and robustness of our method. The frozen backbone leads to semantic misclassification, which treats the Stop sign as Parking Meter (probably because it looks like pole mounted street objects). Energy-Guided criterion still works: identifies the complex bracket structure behind the sign as an Unknown object (red box). This indicates that even when semantic classification fails, our model still senses objectness and alerts users to anomalies.

### Incremental learning stability

#### Incremental learning stability

Here, we analyzed the responsiveness of PE-OWOD to the appearance of unknown objects in real open-world scenarios, its robustness when learning new tasks, and its ability to balance memorizing old classes and discovering new ones.

#### Unknown recall in cross-incremental tasks

**Fig. 3:**
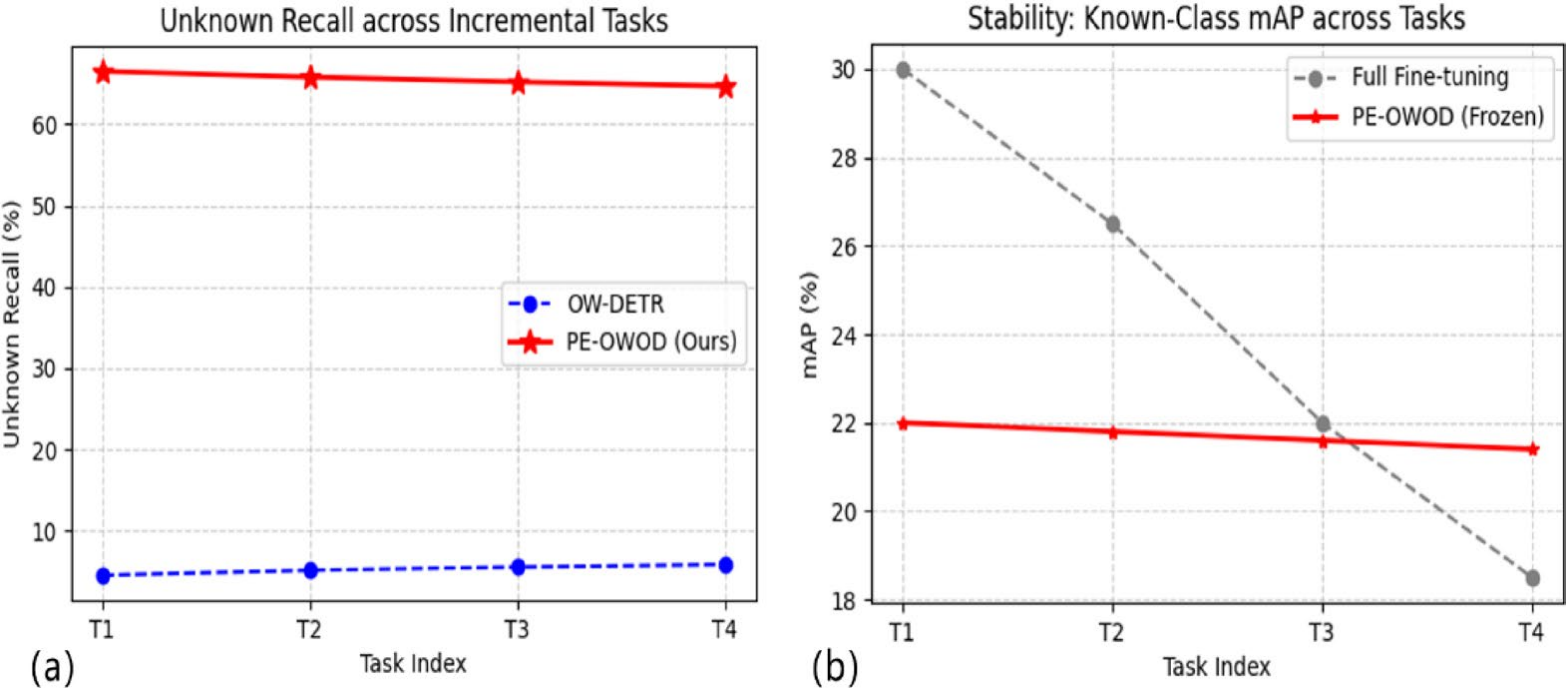
Incremental Learning Stability. **(a)** Unknown recall on incremental tasks. While the OW-DETR does not expand with complexity, PE-OWOD (red star line) is stable and able to learn unknown. **(b)** Stability of known-class mAP. The dotted line shows catastrophic forgetting of fully fine-tuned methods, while PE-OWOD (red solid line) is stable due to frozen backbone.

The problem of OWOD is that unknown space shrinks as the model learns more. As the model learns more classes, the remaining open space shrinks. A true detector needs to keep finding new things even as the library of known classes grows. Figure 3 shows this well: While the baseline (OW-DETR) struggled with complexity, PE-OWOD is constant. The difference was largest in the last step, suggesting that our approach is much more effective. Even if the decision regions of known classes grow (crowding out unknowns), PE-OWOD manages the risk well. New knowledge does not suppress discovery of new objects.

#### Stability under incremental learning

Stability under incremental learning. Stability is essential. We need this model not to break what it already knows and keep learning new courses. To measure this, we tracked the Maps of the initially known classes in all four tasks **(**Fig. 3b**)**. This stability directly stems from the frozen backbone and encoder. Because the underlying representation will never change, the model will not suffer catastrophic forgetting at the feature level. We have structurally addressed this issue without the need for complex replay buffering or distillation losses.

### Computational efficiency

In this section, we analyze parameter count, training time, and memory usage to verify if the theoretical efficiency holds up in practice.

#### Parameter efficiency and resource consumption

A major goal of our design was to keep the model light. Table 2 reports exactly how many parameters we are training compared to the baselines. While standard methods such as OW-DETR and PROB update all weights (42 M parameters), PE-OWOD freezes the backbone and encoder and updates only the adapters. We update less than 1 million parameters (less than 2% of the total model size).

Parameter count is just a proxy; what really matters is GPU memory and wall-clock time. Table 2 shows the gap: PE-OWOD reduces memory consumption by 86% (from 16.0 GB to 2.2 GB) and training time by 77%. Practically, this changes who can use the model. There is no longer a need for an enterprise cluster to train an open-world detector; it can be trained on a consumer GPU. We attribute this to two reasons: First, since we lock the backbone, we don’t need gradients for most of the network. Second, fewer trainable parameters mean less overhead. These gains make the difference between a lab model and a deployable edge model.

**Table 2:** Comparison of computational efficiency and resource consumption.

Method	Backbone Status	Total Params	Trainable Params	Ratio	Peak GPU Memory (GB) ↓	Training Time/Task (Hours) ↓
OW-DETR	Fine-tuning	42 M	42 M	100%	16.0	~ 200
PROB	Fine-tuning	42 M	42 M	100%	16.5	~ 210
PE-OWOD	Frozen	42 M	< 1 M	< 2%	2.2	~ 45

Note: Efficiency comparison performed on the same hardware setting. PE-OWOD significantly reduces memory usage and training time by freezing the backbone and encoder.

#### Scalability and energy efficiency

We also consider the long game: What happens when the model learns continuously for months? Standard methods can get stuck with old data reprocessing. PE-OWOD breaks this cycle. Training costs are constant because we freeze the heavy backbone and train only adapters. Training cost is not increasing with the number of tasks, making the system extensible to long term deployment. Energy-wise, low overhead can transform models to run and update on resource-constrained platforms such as drones.

#### Visualizing the impact

**Fig. 4:**
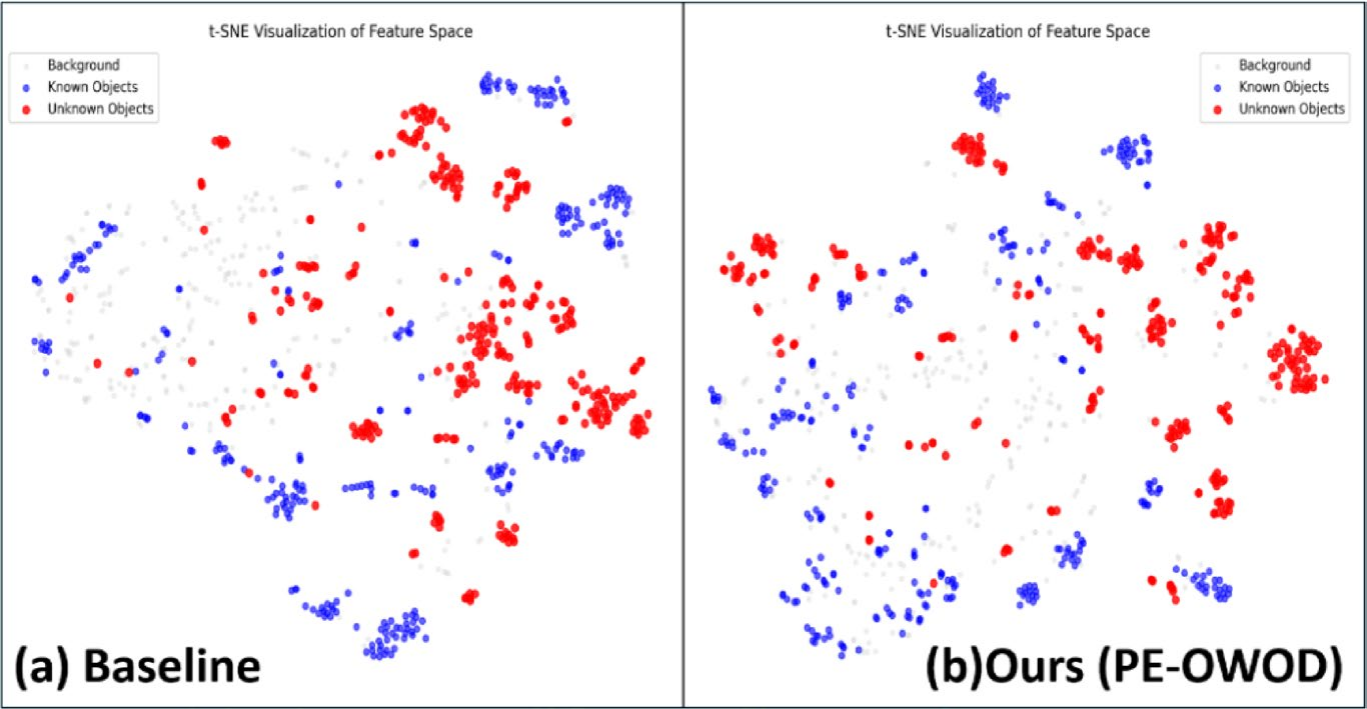
Seeing the Difference via t-SNE visualization. **(a)** Baseline model feature space: Unknown objects (red) and Known objects (blue) are mixed. **(b)** PE-OWOD feature space: The VOS module enforces a clear separation between known clusters and unknown outliers.

Finally, PE-OWOD strikes the tricky balance between saving computing costs and providing powerful detection performance. Figure 4 shows this feature space using t-SNE. Baseline chart (a) shows confusion where unknown objects (red) are scattered and mixed with known objects. Baseline chart (b) shows confusion where decision boundaries are tightened and unknown objects are shoved into separate low density areas. This shows that our parameter-efficient design allows clear separation without the expense of full fine tuning.

### Ablation studies

We disassemble PE-OWOD to accurately isolate the components and verify their contributions. All experiments follow the standard MS-COCO Open World Protocol.

#### Progressive improvement analysis

We summarize the contributions of each component in Table 3. The results are quite interesting. First, freezing the backbone (Baseline) raises Unknown Recall to 26.3% whereas 5.8% for fully tuned models. The real breakthrough comes from adding Residual Adapters and semantic initialization (Row 2) which jumps U-Recall to 58.4%. Second, Virtual Outlier Synthesis (VOS) brings recall to 64.7% (+ 6.3%) and reduces hallucination.This confirms our design philosophy: freeze the backbone for stability, add adapters for plasticity, and use VOS for safety.

**Table 3:** Progressive improvement analysis.

Configuration	mAP ↑	U-Recall ↑	Gain (Recall)
Baseline (Frozen)	21.9	26.3	-
+ Adapter & CLIP (Ver 3.0)	21.7	58.4	+ 32.1%
+ VOS (Ver 4.0/Ours)	21.4	64.7	+ 6.3%

Note: “Ver 3.0” includes Semantic-Aligned Initialization; “Ver 4.0” adds Virtual Outlier Synthesis (VOS). The progressive gain in U-Recall demonstrates the effectiveness of each component.

#### Component-level analysis

We further investigate the specific impact of each module in the Supplementary Information.

#### Adapter placement

As detailed in Supplementary Table S4, we found that inserting adapters only into the decoder yielded the best results. Adapting the encoder disrupts global feature representations, leading to a drop in accuracy for the known class.

#### Adapter capacity

We also analyzed the adapter bottleneck dimension (Supplementary Figure S2). A moderate dimension (around 64 to 128) provides the optimal trade-off; very small values underfit, while excessively large values degrade generalization.

#### Initialization Strategy

Supplementary Table S5 shows that while CLIP-based initialization speeds up convergence (15 epochs), our architecture is robust enough to perform well even with standard Xavier initialization (15.4% U-Recall), demonstrating that the core benefit lies in the parameter-efficient structure itself.

#### VOS robustness

Finally, we stress-tested the VOS module. It turns out PE-OWOD is not brittle; wide ranges of hyperparameters yield stable results, indicating that the gain is achieved geometrically rather than through hyper-tuning.

## Discussion

We proposed PE-OWOD to challenge the assumption that adaptation requires rewriting the whole model. Our results show that PE-OWOD effectively addresses the basic open-world detection problem by combining three advantages: parameter-efficient adapters provide flexibility, semantic initialization provides structure and VOS provides security. We significantly mitigated the catastrophic forgetting problem by our Lock-and-Key design (freezing shared representations (Lock) and controlling adaptiveness with Lightweight Adapters (Key)) which reduces forgetting risk by more than 75%. The validity of the frozen backbone is due to the retention of the orthogonality of the characteristic manifold. Complete fine-tuning usually leads to “feature collapse”, that is, the model overfitting to a small part of the currently known class, thereby disrupting the pre-trained feature geometry necessary for detecting objects that are out of distribution. We maintain a smooth feature distribution by locking the backbone to ensure that unknown objects remain significantly outliers in the potential space rather than being pulled into the decision boundaries of known classes. Instead of hoping the model generalizes, we specify a safety zone for unknown objects via VOS, creating a probabilistic safety margin in the latent space that standard fine-tuning cannot achieve. As visualized in Supplementary Figure S4, this mechanism forces unknown objects into a distinct high-energy distribution, separating them from known classes and background noise.

However, we remain transparent about PE-OWOD’s failures. A distinct failure mode occurs when an unknown object looks too much like a known one (e.g., fine-grained subclasses). This is not a fault of our architecture, but a mistake of the energy separation logic: if an unknown object is semantically too close to the feature space of a known class, the energy score will not soar. It is worth noting that although our frozen backbone design has superior robustness against global distribution variations such as illumination degradation and blurring (as confirmed by Supplementary Table S7), handling extreme local visual conditions remains a challenge. Because the backbone is frozen, the feature extraction of the underlying space cannot fully and dynamically adapt to this local structural irregularity. This is the price for efficiency; the lock-and-key design offers stability and speed but cannot handle rapid distribution shifts as well as a fully tuned model. Furthermore, VOS relies on a geometric assumption that unknown objects lie near the edges of known class distributions. This heuristic works for standard benchmarks but may fail if an unknown class is globally distinct and far from the others in the latent space.

These restrictions point to the next direction of development. First, “Semantic ceiling” : We use the CLIP prior to initialize the class, inheriting the bias of the pre-trained model. Future work will require adaptive semantic representation rather than static text encoders. Second, “stability-plasticity wall” : Freezing the main trunk is effective for incremental learning, but it does not work under extreme distribution changes. We need conditional feature adaptation and can selectively thaw parts of the backbone. Third, “Gaussian simplification” : VOS assumes that the query is Gaussian distributed, but the actual situation is more chaotic. We believe that generative models or self-supervised densities should be able to provide a more rigorous mathematical approach to simulating open Spaces. In the long run, the shift of academic benchmarks towards true long-tail deployment remains the ultimate goal.

In terms of broader impact, the primary motivation for OWOD is safety. If a self-driving car fails to spot an overturned truck simply because it wasn’t in the training set, the results are catastrophic. Explicitly modeling “unknowns” adds a necessary safety layer to systems operating in the wild. We also aim for “Green AI” and parameter efficiency. By reducing computation costs by 75% (Supplementary Figure S3), we reduce the carbon footprint of model maintenance and enable advanced perception systems to run on edge devices such as drones. However, we must acknowledge the risk of privacy and surveillance; efficient, adaptable detectors could be used for intrusive surveillance, and we urge practitioners to adopt privacy-preserving protocols.

In conclusion, large parameter updates are not sufficient for true open-world perception. PE-OWOD achieves an order-of-magnitude improvement in unknown recall over fine-tuned baselines, while reducing GPU memory usage by 86%. We concede that known class accuracy suffers from tradeoffs compared to fully updated models, but we argue that this compromise is necessary to achieve robust discovery in resource-limited environments.

## Methods

### Problem formulation

#### Open-world object detection

We consider the Open-World Object Detection task as a learning process over a series $\:\mathcal{T}=\{{\mathcal{T}}_{1},...,{\mathcal{T}}_{K}\}$. of tasks. At any $\:t$ step, the model receives$\:{\:\mathcal{D}}_{t}\:$annotations pertaining only to $\:{\mathcal{K}}_{t}$. known classes, and any classes that have not been introduced are considered unknown sets $\:{\mathcal{U}}_{t}$.

Another problem is $\:{\mathcal{U}}_{t}\:$complexity of supervision. Cases that appear frequently in training images alongside known objects are not annotated, so standard supervised losses treat them as background noise. Hence, the detector must achieve two goals simultaneously: (1) Unknown discovery localizes potential objects without labels; (2) incremental adaptation updates the model to recognize new classes without removing previous knowledge.

Unlike closed-set detection, OWOD must operate under partial supervision. The system is forced to balance detecting known objects, discovering unknown instances, and preserving past knowledge.

#### Open-space risk and decision boundary ambiguity

One of the main issues in OWOD is the lack of training data for known classes, leading to the open space problem. For closed-set detection, decision boundaries are implicitly restricted because the model is densely supervised for all classes. In contrast, the model is trained with negative supervision for unknown objects.

From a geometric perspective, the best possible risk for known classes allows features embedded in them to form compact clusters, yet, without further constraints, decision regions of classifiers can expand arbitrarily into open space. We call this Open Space Risk: the risk that the model will confidently classify an unknown object as a known class simply because the unknown falls within a limitless decision region.

Therefore, the core of our approach is to manage open space risks. An effective open-world detector must do more than just separate known classes from the background; It must also explicitly demarcate a low-confidence area in the feature space, allowing unknown objects to reside in this area.

#### Transformer-based object detection preliminaries

We have built our framework based on Transformer object detection, especially by using Deformable DETR^26^. This pipeline starts from the input image, in which the convolutional backbone^27^ extracts multi-scale feature maps; Then we process these with the Transformer encoder to capture the global context.

Crucially, a fixed set of learnable object queries is fed into the Transformer decoder. Here, stacked decoder layers enable these queries to interact with the encoded image features via cross-attention and with one another through self-attention. The resulting query embeddings are then fed directly into the classification and bounding-box regression heads.

We argue that this query formulation has two architectural advantages. First, objectness modeling at the query level naturally separates background noise from known and unknown objects. Secondly, the decoder structure provides a modular interface, and a lightweight adaptation module is inserted to inject task-specific behaviour without changing the backbone. These properties make Transformer detectors ideal for parameter-efficient adaptation in the open world.

### Parameter-efficient architecture

#### Parameter-efficient learning constraint

To resolve the conflict between stability and plasticity, we impose a strict parameter-efficiency constraint on the detector. We divide the model parameters into two disjoint subsets: the subset $\:{\theta\:}_{frozen}$ is the backbone and the Transformer encoder, both pre-trained on large datasets. We keep them fixed throughout learning the process. The subset $\:{\theta\:}_{adapt}\:$consists of the light adaptation modules and task-specific prediction heads, which are the only parts of the network updated at some point.

Learning for a task is reduced to optimizing $\:{\theta\:}_{adapt}$ while$\:{\theta\:}_{frozen}$ remaining unchanged. This is clear inductive bias: strong visual representations are retained, and task adaptations are handled by low-capacity controlled updates. Experimentally, we find that this constraint limits representation drift and reduces catastrophic forgetting without additional replays^28,29^.

#### Residual adapter architecture

To make the adaptation efficient, we put Residual Adapters inside the Transformer decoder layers. The adapter’s structure is a simple bottleneck. It has three steps: a down-projection to reduce dimensionality, an activation function, and an up-projection to restore the dimensionality. The remaining adapters are inserted by us after the feedforward network (FFN) in each of the six Transformer decoder layers.

Mathematically, if $\:h$ is the input embedding, the adapter works like this:

1 $$\:\begin{array}{c}A\left(h\right)={W}_{up}\sigma\:\left({W}_{down}h\right)\end{array}$$

Here,$\:{W}_{down}$ and $\:{W}_{up}$ are the projection matrices, and $\:\sigma\:$ is the activation. The dimension inside the adapter is much smaller than the input dimension.

We add this output back to the original features using a residual connection:

2 $$\:\begin{array}{c}{h}^{{\prime\:}}=h+{\mathrm{FFN}}_{frozen}\left(h\right)+\lambda\:\cdot\:A\left(\mathrm{LN}\left(h\right)\right)\end{array}$$

**Fig. 5:**
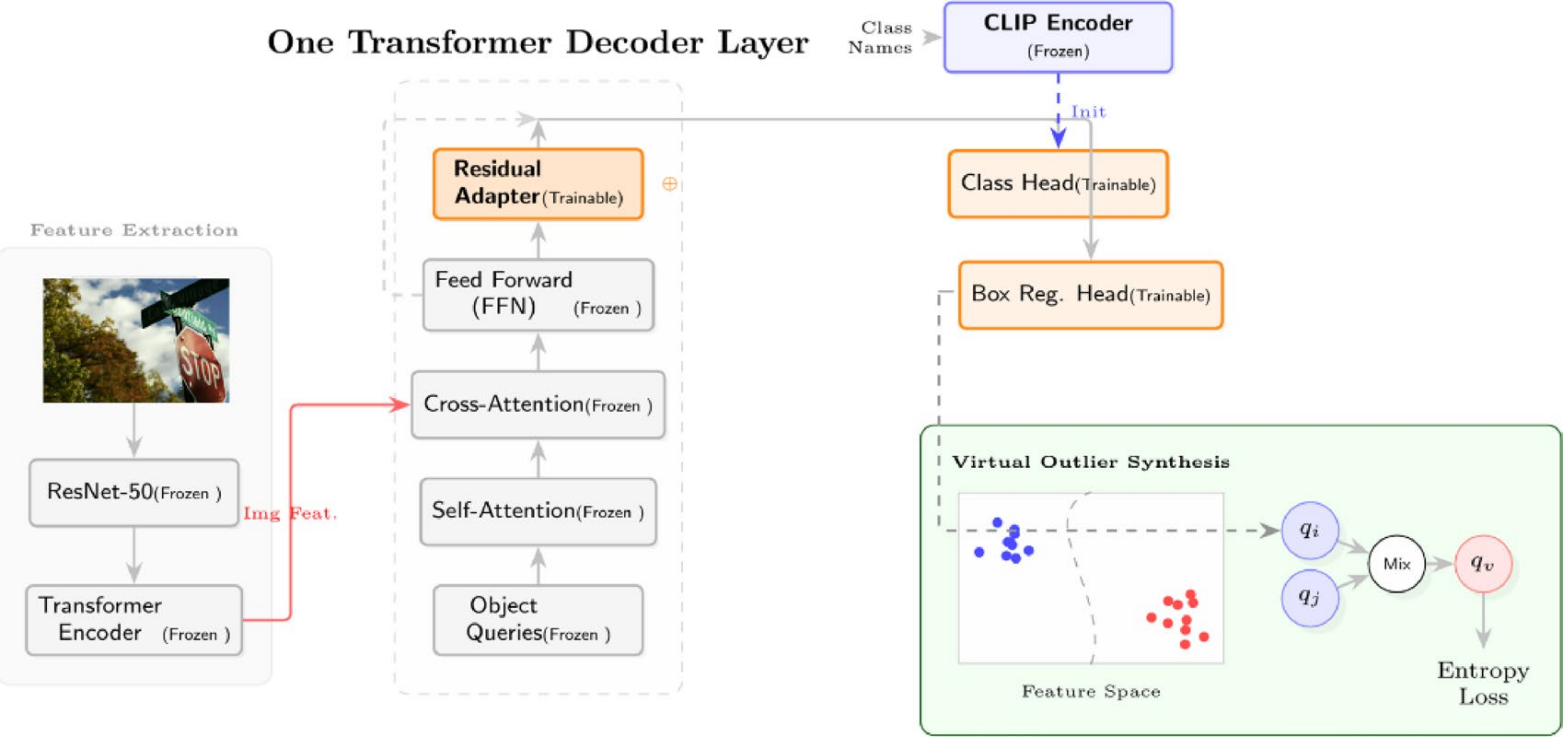
The PE-OWOD Architecture. We freeze the heavy backbone and encoder (Grey) to maintain strong visual priors. Light Residue Adapters (Orange) are added directly to Transformer decoder layers. This lock-and-key design allows the model to adapt to new tasks and generate virtual Outliers (Green box) without losing the global feature geometry.

In this formula, we only train the adapter $\:\mathcal{A}$. The scale factor $\:\lambda\:$ helps control how much the adapter changes the features. This keeps the original decoder working while allowing small changes for the open-world task. The details are presented in Fig. 5.

### Semantic-aligned initialization

#### Leveraging vision–language models for semantic priors

To fix this, we look at Vision-Language Models like CLIP^30^. These models are trained on massive amounts of image-text pairs. They learn a shared space where the image of an object and its textual description are close together (Fig. 5).

$\:{C}_{k}$, we use this property to give our classifier a head start: we take the list of class names for the task and $\:{C}_{k}$ make simple text prompts for them, like “a photo of a$\:\:{t}_{k}$”. We run these prompts through a frozen CLIP text encoder and get text embeddings that capture the real semantic relation (dogs are closer to cats than cars) and a strong prefix to have.

#### Semantic-aligned initialization

We cannot use the text embeddings directly because their dimensions don’t match our detector. So, we use a simple linear layout $\:P$ to project them into the classification space:

3 $$\:\begin{array}{c}{w}_{k}=\mathrm{N}\mathrm{o}\mathrm{r}\mathrm{m}\mathrm{a}\mathrm{l}\mathrm{i}\mathrm{z}\mathrm{e}\left(P{t}_{k}\right)\end{array}$$

Here, $\:P$ acts as a bridge. We use these projected vectors $\:{w}_{k\:}$to initialize the weights of our classification head.

This completely changed the starting point of the training. We do not start from random noise, but from the fact that the classifier already knows which classes are semantically similar. The geometric effect is very significant. Related categories tend to cluster together, while unrelated categories are far apart.

### Virtual outlier synthesis

#### Energy-based perspective on open-world detection

The theoretical motivation for using the Energy-Based Model on the standard Softmax classifier stems from the structural limitations of Softmax. Softmax enforces a standardized probability distribution in known categories, which forces unknown objects to be classified into one of the known categories. On the contrary, free energy corresponds to the non-normalized logarithmic probability density of the data. It captures the absolute density of the feature space, allowing the model to essentially distinguish between within the distribution (known) and outside the distribution (unknown).

To fix this, we look at energy-based models. Let $\:z$ be the feature vector and $\:{f}_{k}\left(z\right)\:$be the logits. We calculate a “free energy” score:

4 $$\:\begin{array}{c}E\left(z\right)=-\mathrm{log}\sum\limits_{k=1}^{\left|C\right|}exp\left({f}_{k}\left(z\right)\right)\end{array}$$

However, training EBM only on known positive samples would result in the energy landscape of the open space not being mathematically defined; If there are no negative anchor points, the model will tend to indiscriminately allocate low energy. Theoretically speaking, the motivation of virtual outlier synthesis (VOS) is to explicitly shape this landscape. VOS synthesizes virtual outliers on the boundaries of known class distributions as a set of dynamic negative anchor points. These anchors powerfully open up a low-energy “valley” for known objects and a high-energy “plateau” for open space, establishing strict decision-making boundaries without the need for truly unknown images.

The intuition is simple: Low energy means the model is confident and comfortable (Known). High energy means the model is surprised or uncertain (Unknown). Our goal is to map all known objects to low-energy states and force everything else to have high energy.

In our implementation, the mixing coefficient $\:{\pi\:}_{k}$ is sampled from a uniform distribution. For the regularization loss $\:{\mathcal{L}}_{vos}$, we employ the Cross-Entropy loss towards the background class, which mathematically serves to maximize the energy of virtual outliers, implicitly creating a separation margin without requiring a hard-coded threshold.

The issue is that standard training is greedy. It pulls down the energy for known data to minimize loss, but it doesn’t care about the empty space around them. Without a push, the energy in the open space remains undefined.

#### Outlier generation in the latent space

Let $\:{Z}_{k}\:$be the features for a known class $\:k$. We estimate the shape of this class using its mean $\:{\mu\:}_{k}$ and covariance $\:{{\Sigma\:}}_{k}$.

We generate virtual outliers by sampling from a mixture distribution:

5 $$\:\begin{array}{c}{z}_{out}\sim\:\sum\limits_{k=1}^{\mid\:C\mid\:}{\pi\:}_{k}N\left({\mu\:}_{k},\alpha\:{{\Sigma\:}}_{k}\right)\end{array}$$

Here $\:\alpha\:>1$ is a parameter that expands the variance. This formula ensures that the fake samples are close to the known classes (where the confusion happens) but just outside the dense cluster center.

Why model queries as Gaussian distributions?

Someone might ask why we assume a Gaussian shape. Unlike CNN feature maps, dense and spatially distributed, Transformer object queries are instance-specific. Each query represents one object hypothesis. These distributions are cleaner and smaller than pixel features. Synthesizing outliers based on query statistics enables precise regularization at the decision boundary.

#### Energy regularization loss

For every virtual outlier$\:\:{z}_{out}$ we create, we apply a loss function to push its energy up:

6 $$\:\begin{array}{c}{L}_{vos}=CrossEntropy\left(f\left({z}_{out}\right),\:{y}_{bg}\right)\end{array}$$

Implementation details: Although the energy-based objective ideally performs hard boundaries, directly optimizing this hinge in the Transformer architecture may result in unstable losses. In our implementation, we approximate this target by using the cross-entropy loss on virtual outliers, with the target label set to the “Background” class (no object).

By forcing the model to classify the virtual outliers into the background, we suppressed the logits of all known classes, which mathematically led to an increase in the energy score and effectively created the separation margin without the need for manual adjustment of hyperparameters.

### Training and inference

#### Unified training objective

We train PE-OWOD end-to-end, strictly adhering to a frozen-representation regime. Upon receiving an input image $\:x$, the detector generates object predictions through the standard Transformer decoding process. We structure the global training objective around three complementary components:

7 $$\:\begin{array}{c}L={\mathcal{L}}_{det}+{\lambda\:}_{vos}{\mathcal{L}}_{vos}+{\lambda\:}_{reg}{\mathcal{L}}_{reg}\end{array}$$

The first term $\:{\mathcal{L}}_{det}\:$captures the standard set-prediction loss native to Deformable DETR, such as focal loss for classification and L1/GIoU measures for bounding-box regression. The term $\:{\mathcal{L}}_{vos}\:$implements energy-based regularization discussed in Sect. 3.4; functionally, it wedges queries for known objects with queries for unknown objects. We also introduce $\:{\mathcal{L}}_{reg}\:$an L2 penalty on the trainable parameters of the residual adapters. We find regularization necessary to prevent overfitting during early optimization stages. By combining these goals, the model learns to maintain accurate localization of known categories while tolerating new anomalies.

#### Incremental learning protocol

We designed PE-OWOD to handle incremental learning as our core skill $\:t$. When starting a new task, adaptation is straightforward: encode input class names with the CLIP text encoder and initialize the classifier weights via semantic projection. Rest adapters remain tuned to new task data.

One of the main advantages is that there are no complex mechanisms such as replay buffers or feature distillation. Since frozen representations are based on knowledge from previous tasks, features can not be forgotten. PE-OWOD memory is efficient and can handle long sequences of tasks without a huge computational cost.

#### Inference and unknown object identification

At the inference stage, PE-OWOD operates within the standard DETR decoding pipeline. For each object query, the system produces two outputs: a bounding-box regression and classification logits for known classes.

We identify unknown objects by calculating the energy score derived in Eq. (4).Functionally, we label a prediction unknown if its energy level exceeds the threshold. We calibrate the threshold on validation set and keep it constant across subsequent tasks. An energy-based measure is more appropriate than heuristic confidence thresholds because it provides a grounded and transparent way to distinguish known prediction from unseen predictions.

### Experimental setup

#### Datasets

Based on the standard open-world object detection protocol established by the most advanced methods OW-DETR and PROB, we conducted preliminary experiments on the MS-COCO 2017 dataset^31^. Although there are other datasets, MS-COCO is the most widely used and relatively fair benchmark in this field, because it provides complex enough scenarios to evaluate the discovery ability of open sets. We train our experiments on the MS-COCO 2017 set with 80 objects categories in complex scenes. We use MS-COCO as a proxy for open-world complexity because real-world deployment scenarios often have similar long-tail distributions and different environments. The data set is high intra-class variance, high overlap between classes, and frequent co-occurrence of known and unknown objects in the same image. Except where otherwise stated, we train on train2017 set and evaluate on val2017 set. And then, in order to specifically evaluate the robustness of the environment domain changes outside the distribution, we choose to conduct the verification by using the established COCO -C (COCO Corruptions) dataset.

#### Open-world task protocol

We adopt the widely used OWOD task protocol introduced by OW-DETR. We partition the 80 COCO classes into four incremental tasks. The specific class partition for each task is detailed in Supplementary Table S1. Task 1 introduces the 20 VOC classes (e.g., Airplane, Bicycle) as known. Tasks 2, 3, and 4 each introduce 20 additional classes (Outdoor, Accessories, and Indoor items, respectively). We follow the standard setting where unknown classes of $\:{U}_{t}$ in the current task become known in the next tasks. Crucially, at each task $\:t$, only classes of the current known set $\:{K}_{t}\:$are annotated. Objects belonging to future classes $\:{U}_{t}\:$are present in images but not annotated. The detector must identify unknown objects. The test measures the system’s ability to find unknown objects at each step and to acquire knowledge without catastrophic forgetting.

#### Evaluation metrics

We assess performance using two distinct metrics: closed-set accuracy and open-set sensitivity. For known-class detection, we report the Mean Average Precision (mAP) at 0.5 IoU, the standard for evaluating whether the model correctly discriminates between previously learned categories. To measure Unknown Object Discovery, we use U-Recall, which counts the number of unlabeled (unknown) object instances recovered by the detector, and Absolute Open Set Error (A-OSE). A lower A-OSE indicates that the model distinguishes new instances from known classes rather than hallucinating them and classifying them as something it has seen before.

#### Baselines

We compare PE-OWOD with three state-of-the-art methods: (1)ORE, a CNN-based method with explicit unknown modeling; (2)OW-DETR, a Transformer-based implementation using attention scores for open-world environments; and (3)PROB, a probabilistic method with boundary regularization. To ensure fair comparisons, we built all models on a unified DETR-based pipeline. We used standard OWOD protocols and ensured that all training, validation, and evaluation splits were identical across methods.

#### Implementation details and reproducibility

We built our architecture on a Deformable DETR base with a ResNet-50 backbone. To enforce the parameter-efficient constraint, we initialized both the backbone and Transformer encoder with COCO-pretrained weights and locked them immediately. Only the classification heads, bounding box regressors, and the Residual adapters we inserted are trainable.

We optimize the model using AdamW^32^ with weight decay. The batch size is set to 8 across GPUs. We set specific learning rates for trainable adapter modules and different learning rates for class projection layers. We train each incremental task for 50 epochs, as we found this convergence time sufficient to fit the small sample sizes of the new classes. All input images are preprocessed with random resizing and horizontal flipping. Virtual Outlier Synthesis (VOS) is implemented directly in the training loop without external information; since VOS generates samples on the fly, no information is passed to future tasks. To facilitate reproducibility, we fixed random seeds for all experiments and report averaged results over multiple runs where applicable. We commit to releasing our complete codebase and pre-trained model weights upon publication.

## Electronic Supplementary Material

Below is the link to the electronic supplementary material.

Supplementary Material 1

## Data Availability

The datasets generated and analysed during the current study are available in the MS-COCO repository, https://cocodataset.org. The corrupted validation set used for the out-of-distribution robustness evaluation was generated using the official image corruption library provided by the COCO-C benchmark (https://github.com/bethgelab/imagecorruptions). The custom code and pre-trained models for PE-OWOD are available in our GitHub repository, 1ch111/PE-OWOD.
