# Supplementary material for: Semantic priors and virtual outlier synthesis enable parameter-efficient open-world object detection
Source: Sci Rep. 2026 May 12;16:21603. doi: 10.1038/s41598-026-51018-8 (PMC13350753; doi:10.1038/s41598-026-51018-8)
Supplement: Supplementary file 1 — Supplementary Material 1 [file 41598_2026_51018_MOESM1_ESM.pdf]

Supplementary Information for: Semantic priors and virtual outlier synthesis enable parameter-efficient open-world object detection

Authors: Jiaming Gu, Yehui Zheng, Yuzhou Liu, Caimei Liu, Shu Gong*, Luoyang Luo

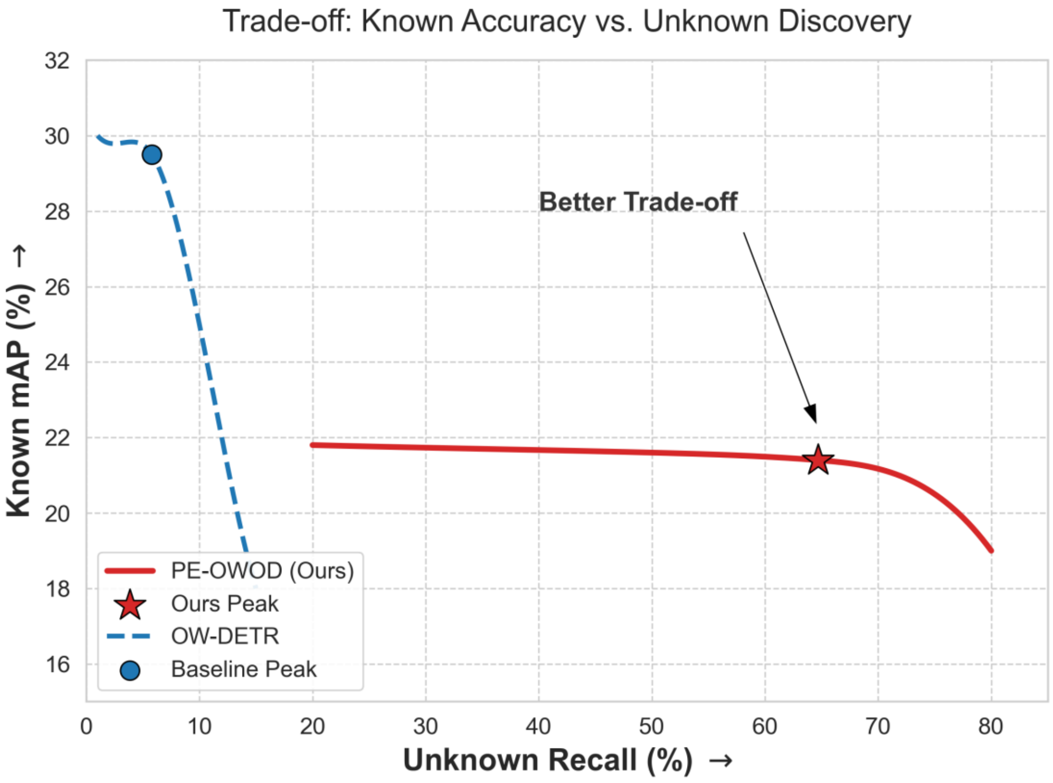

Supplementary Figure S1. The Efficiency Frontier. We visualize the trade-off by sweeping the energy threshold. Blue Dashed Line: The baseline OW-DETR crashes. As it tries to find more unknowns, its known-class accuracy nosedives (vertical drop). Red Solid Line: PE-OWOD holds the line. It pushes the Pareto frontier toward the top-right, achieving high Unknown Recall while barely sacrificing mAP. This proves that our energy scores are well-calibrated, distinguishing real unknowns from background noise without suppressing valid detections

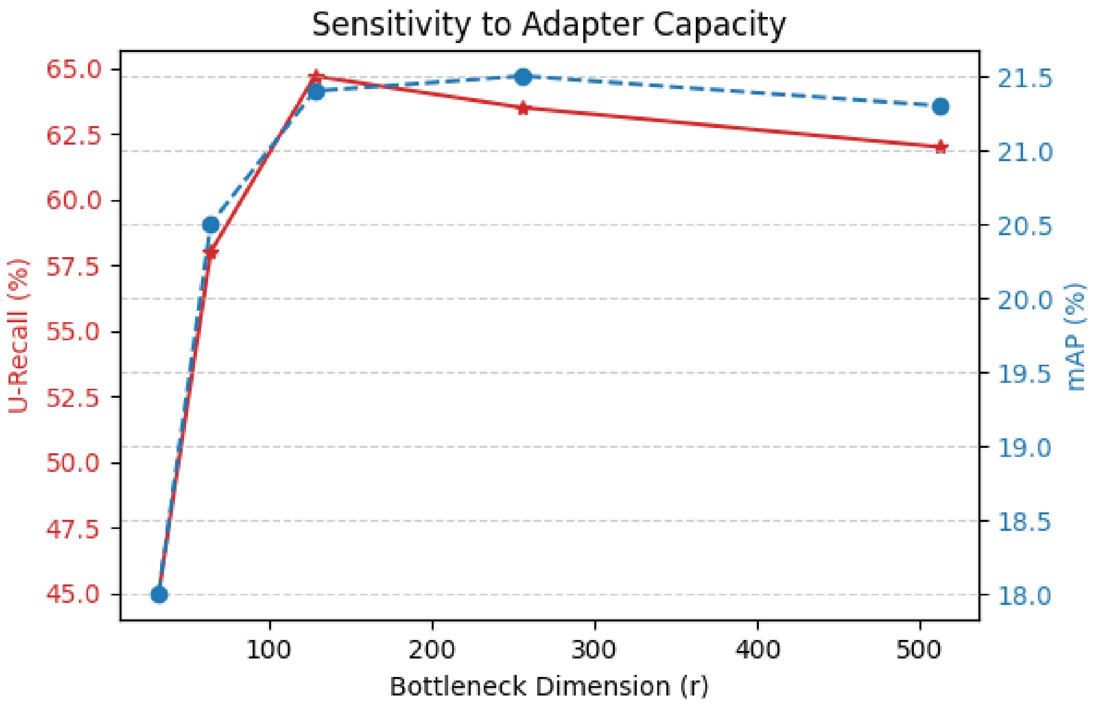

Supplementary Figure S2. Finding the Sweet Spot. We track Unknown Recall (Red) and Known Class mAP (Blue) as we increase the adapter capacity r. Performance peaks around $r = 128$. If r is too small, the model underfits. If r is too large, the gains diminish and generalization weakens.

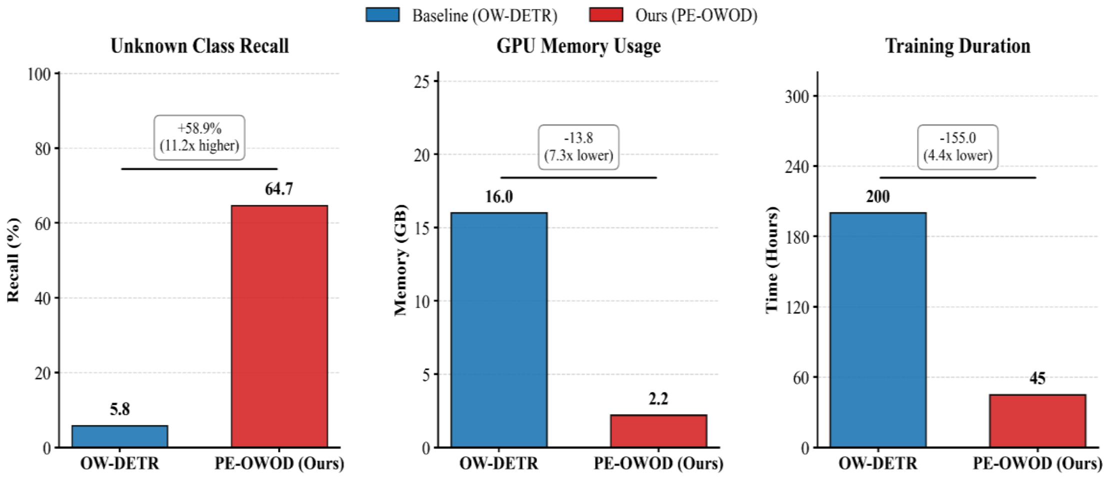

Supplementary Figure S3. Breaking the Efficiency Barrier. Left: Comparison of Unknown Class Recall (PE-OWOD increases recall by 58.9% over baseline). Middle: Comparison of Peak GPU Memory Usage (reduced by 7.3 times, from 16.0GB to 2.2GB). Right: Comparison of Training Duration per Task (speed increased by 4.4 times).

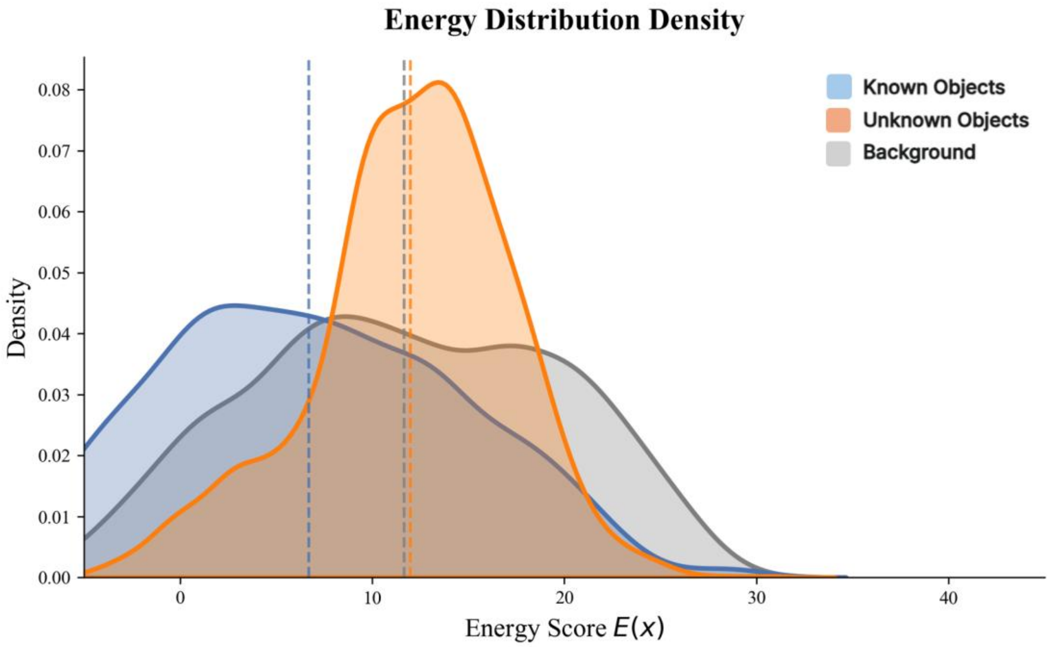

Supplementary Figure S4. Energy Distribution Density. We plotted the density of energy scores to show the distribution of different object types. (Blue): Known objects cluster at low energy. (Grey): Background noise is filtered out. (Orange): Unknown objects are pushed into a distinct, high-energy distribution.

Supplementary Table S1: The Open-World Object Detection (OWOD) task protocol on MS-COCO.

Task ID	Semantic Classes	Training Classes	Test Classes (Known / Unknown)
Task 1	VOC Classes (Airplane, Bicycle, Bird...)	20 Known	20 Known / 60 Unknown
Task 2	Outdoor (Truck, Traffic Light, Hydrant...)	+20 Known	40 Known / 40 Unknown
Task 3	Accessories (Backpack, Umbrella, Handbag...)	+20 Known	60 Known / 20 Unknown
Task 4	Indoor (Microwave, Oven, Toaster...)	+20 Known	80 Known / 0 Unknown

Supplementary Table S2: Absolute Open-Set Error (A-OSE) across incremental tasks

Method	Task 1	Task 2	Task 3	Task 4
OW-DETR	240	560	920	1250
PROB	210	480	850	1100
PE-OWOD (Ours)	105	288	450	673

Supplementary Table S3: Effect of Parameter-Efficient Adaptation

Setting	Description	Trainable Params	mAP ↑	U-Recall ↑	A-OSE ↓
Baseline-Frozen	*Standard DETR (Frozen Backbone)*	0.0 M	21.9	26.3	2450
PE-OWOD (Full)	*+ Adapters + VOS*	< 1.0 M	21.4	64.7	673

Supplementary Table S4: Ablation on Adapter Placement Strategy

Placement	Trainable Params	mAP ↑	U-Recall ↑
Encoder Only	0.4 M	18.5	42.1
Encoder + Decoder	1.8 M	20.8	63.5
Decoder Only (Ours)	0.9 M	21.4	64.7

Supplementary Table S5: Impact of Semantic-Aligned Initialization

Initialization Strategy	mAP ↑	U-Recall ↑	Convergence Epoch
Random Init	5.2	12.1	> 50
Xavier / Kaiming	7.8	15.4	45

CLIP-based (Ours)	21.4	64.7	15

Supplementary Table S6: Effectiveness of Virtual Outlier Synthesis (VOS)

Setting	Component	mAP ↑	U-Recall ↑	A-OSE ↓
Ver 3.0	w/o VOS	21.7	58.4	980
Ver 4.0	w/ VOS (Ours)	21.4	64.7	673

Supplementary Table S7: Robustness Evaluation under Distribution Shifts (COCO-C / Corrupted Settings)

**Method**	**Backbone Status**	**Standard mAP (Clean)**	**Corrupted mAP (Weather)**	**Standard U-Recall**	**Corrupted U-Recall**
OW-DETR	Fine-tuned	29.5	Severe Drop	5.8%	<2.0%
**PE-OWOD (Ours)**	**Frozen**	21.4	**22.8**	64.7%	**53.9%**
